# The GA and ABA signaling is required for hydrogen-mediated seed germination in wax gourd

**DOI:** 10.1186/s12870-024-05193-3

**Published:** 2024-06-13

**Authors:** Jingjing Chang, Jiawei Li, Jinlong Li, Xiao Chen, Jiabin Jiao, Jing Li, Zhao Song, Baige Zhang

**Affiliations:** Key Laboratory for New Technology Research of Vegetable, Vegetable Research Institute, Guangdong Academy of Agricultural Sciences, Guangzhou, 510640 China

**Keywords:** Seed germination, Hydrogen-rich water (HRW), Gibberellin, Abscisic acid, Wax Gourd

## Abstract

**Background:**

Hydrogen gas (H_2_), a novel and beneficial gaseous molecule, plays a significant role in plant growth and development processes. Hydrogen-rich water (HRW) is regarded as a safe and easily available way to study the physiological effects of H_2_ on plants. Several recent research has shown that HRW attenuates stress-induced seed germination inhibition; however, the underlying modes of HRW on seed germination remain obscure under non-stress condition.

**Results:**

In this current study, we investigated the possible roles of gibberellin (GA) and abscisic acid (ABA) in HRW-regulated seed germination in wax gourd (*Benincasa hispida*) through pharmacological, physiological, and transcriptome approaches. The results showed that HRW application at an optimal dose (50% HRW) significantly promoted seed germination and shortened the average germination time (AGT). Subsequent results suggested that 50% HRW treatment stimulated GA production by regulating GA biosynthesis genes (*BhiGA3ox*, *BhiGA2ox*, and *BhiKAO*), whereas it had no effect on the content of ABA and the expression of its biosynthesis (*BhiNCED6*) and catabolism genes (*BhiCYP707A2*) but decreased the expression of ABA receptor gene (*BhiPYL*). In addition, inhibition of GA production by paclobutrazol (PAC) could block the HRW-mediated germination. Treatment with ABA could hinder HRW-mediated seed germination and the ABA biosynthesis inhibitor sodium tungstate (ST) could recover the function of HRW. Furthermore, RNA-seq analysis revealed that, in the presence of GA or ABA, an abundance of genes involved in GA, ABA, and ethylene signal sensing and transduction might involve in HRW-regulated germination.

**Conclusions:**

This study portrays insights into the mechanism of HRW-mediated seed germination, suggesting that HRW can regulate the balance between GA and ABA to mediate seed germination through ethylene signals in wax gourd.

**Supplementary Information:**

The online version contains supplementary material available at 10.1186/s12870-024-05193-3.

## Background

Wax gourd (*Benincasa hispida*), also known as white pumpkin, white gourd, Chinese preserving melon, and ash gourd, is an important species in Cucurbitaceae family that originates from the Indo-China region [1]. Due to its high resistance to the environmental stress, excellent storage capacity, high yield, and low planting cost, wax gourd is one of the main varieties of Cucurbitaceae vegetables in the tropical and subtropical regions of Asia [2]. Furthermore, it plays a significant role in ensuring the annual supply of vegetables and regulating off-seasons, making it a popular vegetable crop worldwide [3, 4]. However, the seed coat of wax gourd is thick and hard, and rich in cuticle, which leads to poor water permeability and absorption, and is one of the key factors affecting seed germination. Additionally, the cotyledons of wax gourd contain substances that affect metabolic activities of seed embryos, such as fat, citrulline, and saponins, resulting in low germination rate and slow [5]. The germination rate of commercial seeds of wax gourd is only 60%, which is lower than that of other crops, increasing the seed cost of melon farmers and seedling enterprises. Therefore, it is of practical significance to solve the problems of slow germination, low germination rate and irregular emergence of wax gourd seeds to promote standardized seedling raising, ensure annual seedlings supply, and improve the economic benefits of farmers and seedling enterprises.

Seed germination is a critical phase in the life cycle of higher plants and controlled by a series of internal (phytohormones) and external (temperature, light, water, etc.) factors [6]. Plentiful research has shown that hormonal signals, especially abscisic acid (ABA) and gibberellin (GA), play a crucial role in regulating seed dormancy and germination. The ABA metabolism and signaling pathway promote seed dormancy and negatively regulate seed germination [7, 8]. It has been demonstrated that ABA biosynthesis genes (e.g., *NCED*), receptors (PYR/PYL/RCAR), SnRK2 protein kinases, and ABA-insensitive factors (such as *ABI3*, *ABI4*, and *ABI5*) are negative regulators of the seed germination pathway, while PP2Cs protein phosphatases act as positive regulators [9–14]. Conversely, GA plays a positive regulation role in seed germination, which has an antagonistic relationship with ABA [6, 8, 15, 16]. In GA signaling pathway, DELLA proteins are key repressors that interact with various transcription factors to inhibit or release GA signaling, thus controlling seed germination [17, 18]. For example, some DELLA proteins interact with ABI5 to establish the crosstalk between GA and ABA signaling [16, 19, 20]. In addition to GA and ABA, ethylene (ETH) also plays a key role in the dormancy-breakage and germination, mainly through working synergistically with GAs but antagonistically with ABA [21, 22]. Linkies et al. postulated a model for the action of ETH and interaction with GA and ABA during seed germination, that is, high GA and ETH sensitivity is a prerequisite for the ethylene-enhanced expression of genes that are involved in endosperm weakening and inhibited by ABA [23].

Hydrogen (H_2_), the simplest gaseous molecule in nature, has been demonstrated to have important biological effects on plant physiological and developmental processes and to play a positive role in counteracting oxidative stress induced by biotic and abiotic stimuli. Accumulated evidence demonstrates that H_2_ is an effective antioxidant that scavenges reactive oxygen species (ROS) by increasing the activity of antioxidant enzymes and restoring redox homeostasis [24–26]. In addition, it has been found that H_2_ plays active roles in regulating several plant growth and development stages, such as seed germination, root morphogenesis, stomatal closure, and fresh keeping of fruits [27–30]. Notably, the function of H_2_ in plant was first reported to induce seed germination in winter rye [31]. Several recent studies have proved that H_2_ accelerates the physiological process of seed germination in plants. For example, pretreatment with hydrogen-rich water (HRW, a H_2_ donor) attenuated the inhibition of seed germination caused by salt and drought stress [27, 32]. Another research found that H_2_ pretreatment alleviated aluminum (Al)-induced germination inhibition by reestablishing the balance between GA and ABA in rice [33]. Numerous studies have mainly focused on investigating the mechanism associated with its responses to seed germination under abiotic stress, while the possible roles of H_2_ in breaking seed dormancy and promoting germination under normal condition is still an interesting and obscure subject. Meanwhile, the understanding of interaction between HRW and hormone signaling pathways, such GA and ABA on seed germination need to be further elucidated.

In this report, we aimed to screen the effective concentration of HRW for seed immersion and investigate the functions of GA and ABA in the regulation of HRW on the germination of wax gourd seed through inhibitor treatments (PAC and ST, respectively). Furthermore, we also explored the potential effects and underlying mechanisms of HRW in seed germination in the presence of GA or ABA using high-throughput mRNA sequencing analysis. Our findings suggest that GA and ABA signals are necessary for HRW to influence seed germination in wax gourd. Such a mechanism of H_2_ has significant implications for its application in agricultural, particularly in combination with plant growth regulators.

## Results

### HRW improved seed germination of wax gourd in a dose-dependent manner

**Fig. 1:**
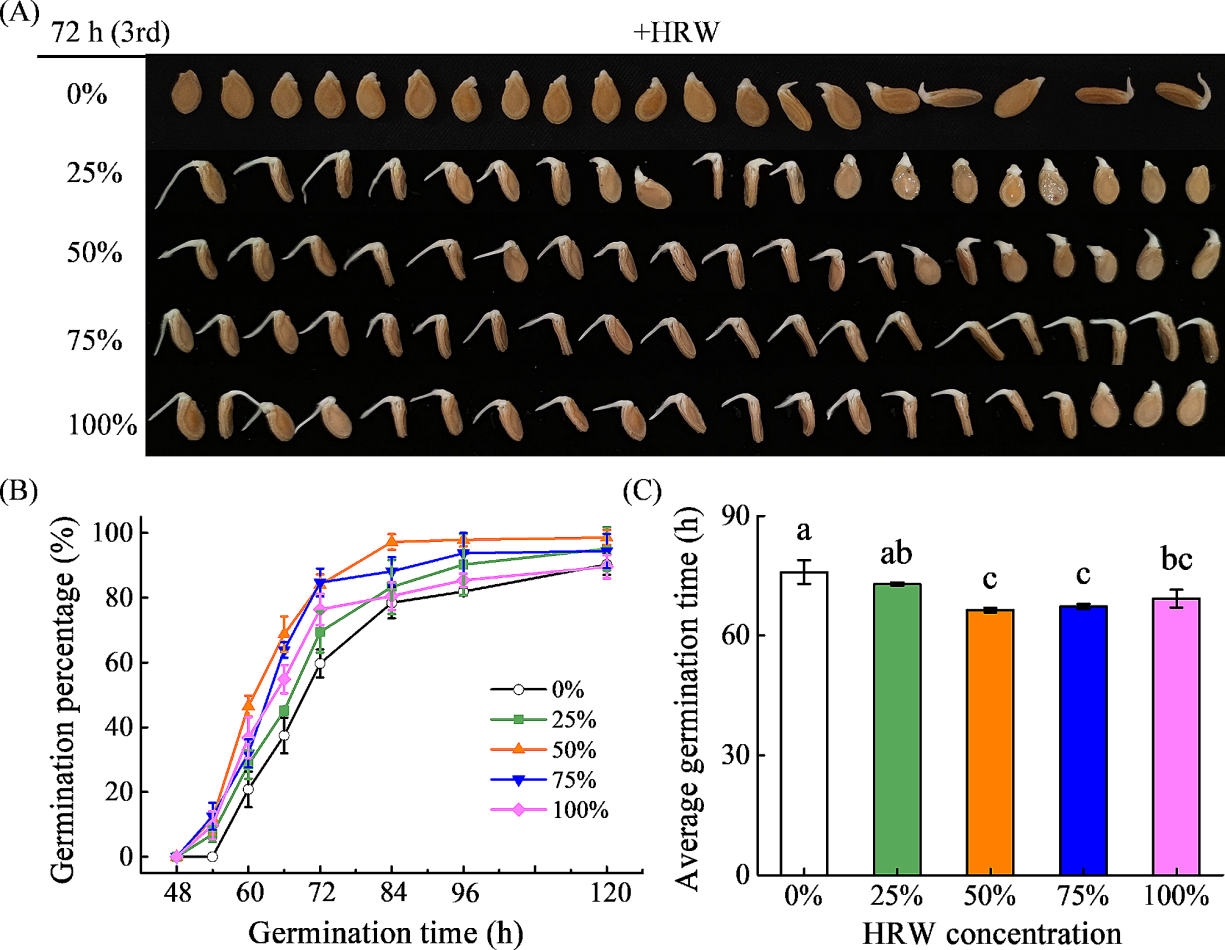
Effects of different concentrations of HRW treatment on seed germination percentage (**A**, **B**) and average germination time (**C**) in wax gourd. Seed were presoaked in HRW solutions of varying concentrations for 6 h and then transferred to an incubator at 30℃ for 120 h (5 days). Photographs were taken at 72 h. The H_2_ concentrations of 0%, 25%, 50%, 75%, and 100% HRW solution are 0, 0.315, 0.630, 0.945, and 1.260 mg L^− 1^.The data are presented as mean ± standard deviation (SD). Different letters above the means denote a significant difference at *P* < 0.05 according to Turkey’s test

To investigate the effect of H_2_ on seed germination, wax gourd seeds were immersed in different concentrations of HRW (0, 25, 50, 75, and 100%). Compared with the control (0%), treatments with 25%, 50%, 75%, and 100% HRW improved the seed germination quality and percentage in different degrees (Fig. 1). The HRW treated seeds began to germinate at 54 h, which was 6 h earlier than that of the control. The 50% germination time (G50) of 50% HRW, 75% HRW, and 100% HRW treatment was between 60 and 66 h of incubation, while that of 25% HRW and CK treatment was between 66 and 72 h (Fig. 1B). At 66 h of incubation, the germination percentages of seeds treated with control and 25−100% HRW were 37.5%, 45.1%, 68.8%, 63.9%, and 54.9%, respectively. Among them, the optimum concentration range of HRW was 50–75%, the germination percentages of which were significantly higher than that of the control at all measuring time points. For example, the germination percentages of seeds treated with 50% and 75% HRW at 72 h after imbibition were 84.0% and 84.7%, respectively, which were significantly higher than that of control (59.7%). The average germination time (AGT) reflects the uniformity and consistency of seed germination during the whole process. In this experiment, the AGTs were shortened to varying degrees by HRW treatment with four concentrations, in which the AGT of 50% HRW treatment was shorten 9.5 h compared with the control (Fig. 1C). Therefore, the 50% HRW treatment was used for further studies during the seed germination process.

### HRW increases GA/ABA ratio by promoting GA biosynthesis

**Fig. 2:**
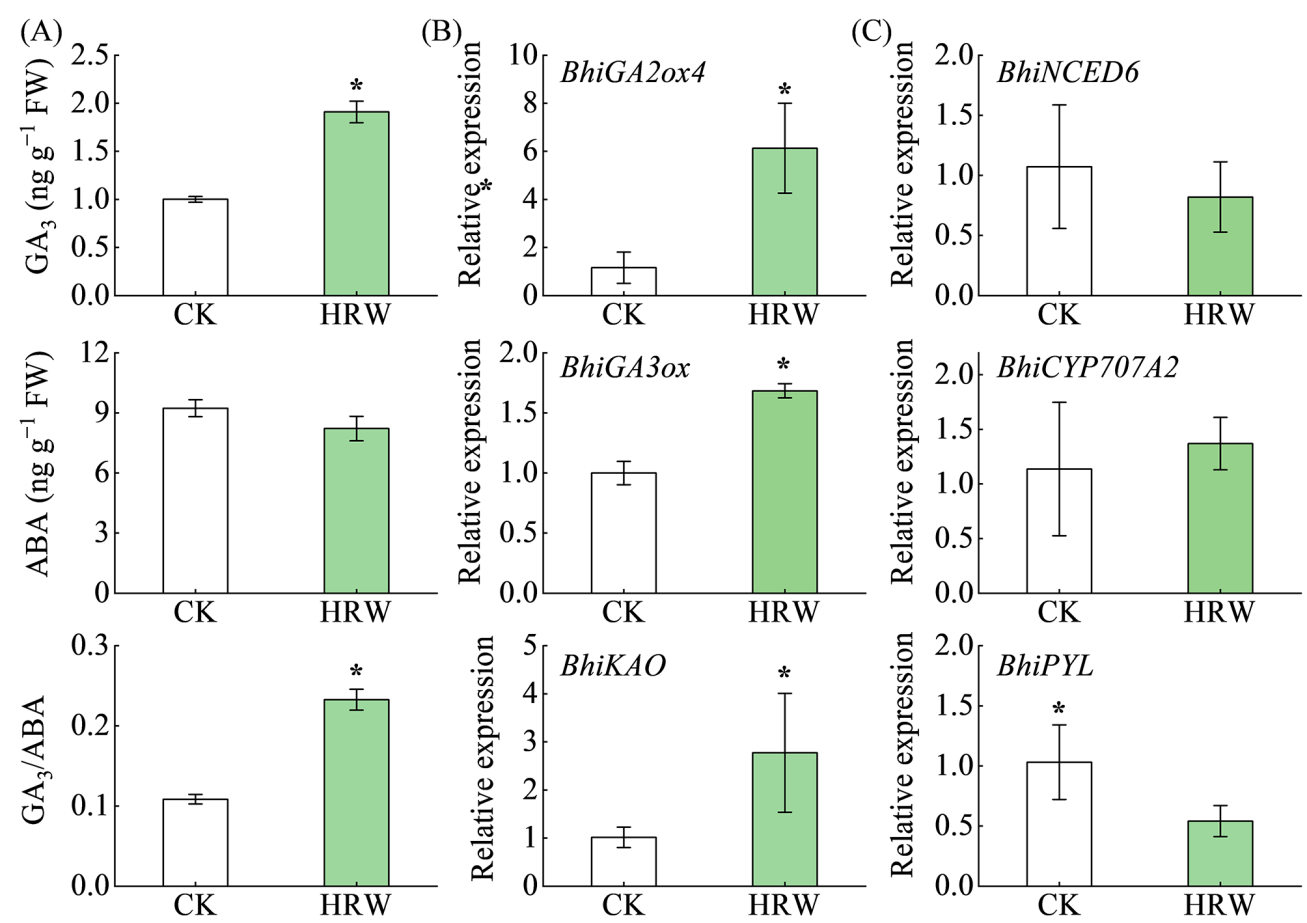
Effects of HRW application on GA/ABA metabolism in germinating seeds. Seeds were presoaked in distilled water (CK) or 50% HRW (HRW) for 6 h and transferred into an incubator at 30℃ for 48 h. (**A**) showed the contents of GA_3_ and ABA and the ratio of GA_3_/ABA. (**B**, **C**) indicated the expression analysis of GA biosynthesis and ABA metabolism genes via qRT-PCR in germinating seeds treated with or without HRW, respectively. The data are presented as mean ± standard deviation (SD). Asterisks (*) represent significant differences between CK and HRW treatments according to *t*-test (*P* < 0.05)

In this present study, to investigate whether GA and ABA might participate in H_2_-induced seed germination, GA and ABA contents were measured after 48 h of seed incubation. Relative to control (CK), HRW treatment drastically promoted the contents of GA_3_, increasing by 91.0%, but had no significant effect on ABA content, resulting in an increase in the ratio of GA_3_ and ABA (Fig. 2A). Subsequently, the transcript levels of GA biosynthesis genes and ABA metabolism genes were determined. The genes related to GA synthesis and ABA metabolism were obtained by alignment with their homologous genes in other species. The six genes (*BhiGA3ox*, *BhiGA2ox4*, *BhiKAO*, *BhiNCED6*, *BhiCYP707A2*, and *BhiPYL*) were selected based on phylogenetic analysis. Compared to CK, HRW treatment increased the transcript levels of *BhiGA2ox4*, *BhiGA3ox*, and *BhiKAO* by 5.29, 1.68, and 2.73 folds, respectively. However, there was no significant difference in the transcripts of *BhiNCED6* and *BhiCYP707A2* between the CK and HRW treatment, while the transcript of *BhiPYL* (an ABA receptor gene) was decreased by 1.90 folds in HRW-treated seeds (Fig. 2B, C). These results suggested that HRW increased the content of GA mainly by promoting the expression of GA biosynthesis genes during seed germination, which in turn raised the GA/ABA ratio. Additionally, HRW-induced seed germination may occur through increasing GA content and inhibiting the ABA signal pathway.

### Involvement of GA in HRW-mediated seed germination in wax gourd

**Fig. 3:**
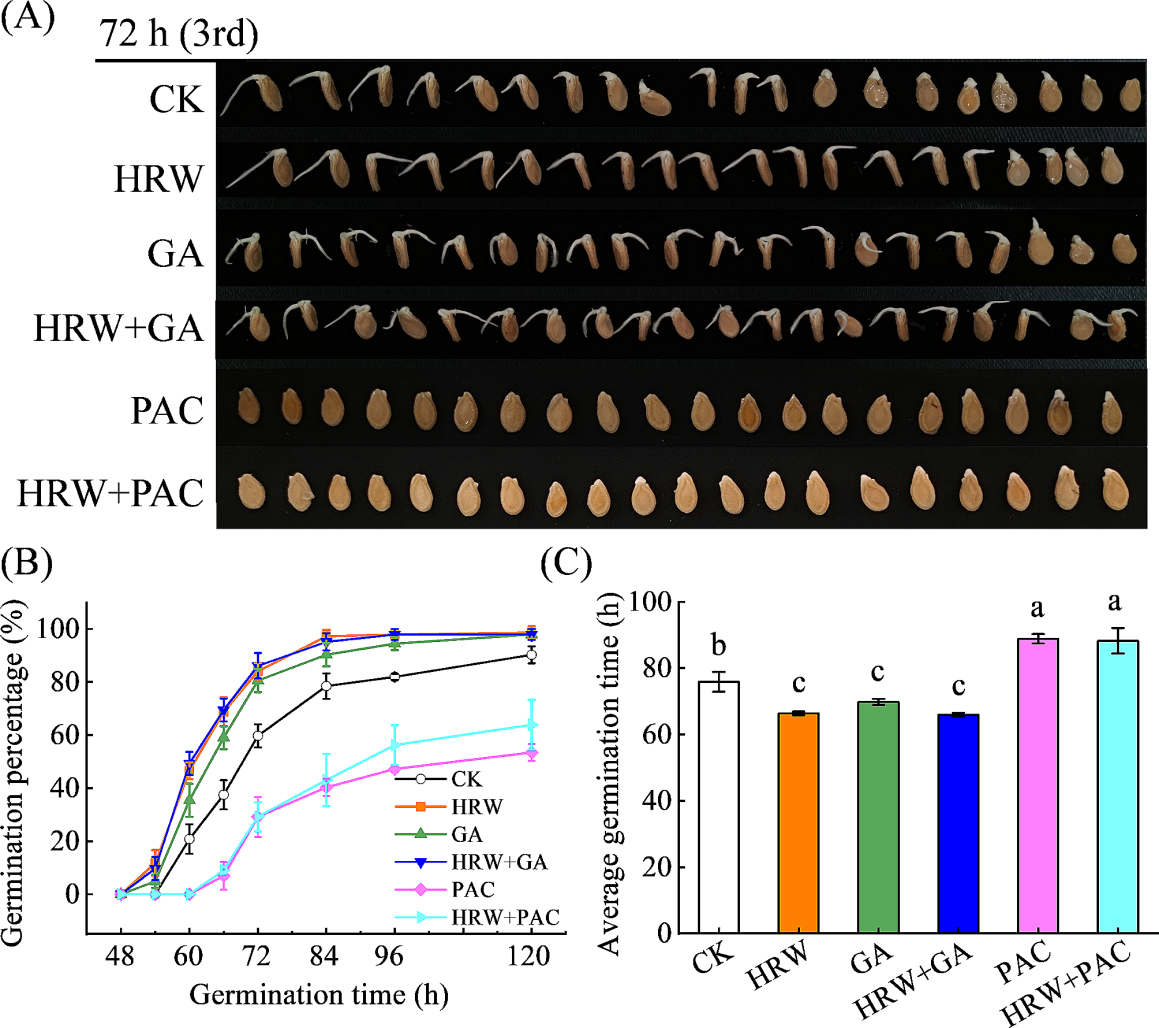
The role of GA in HRW-mediated seed germination in wax gourd. Seeds were presoaked in distilled water (CK) or 0.9 mM GA_3_ solution with or without 50% HRW (HRW). To inhibit GA production, seeds were pre-incubated in 0.3 mM paclobutrazol (PAC) in the presence or absence of 50% HRW. After immersion for 6 h, seeds were transferred into an incubator at 30℃ for 120 hours (5 days). The photographs were taken at 72 hours (**A**). Meanwhile, seed germination percentage (**B**) and average germination time (**C**) were determined. Seeds immersed with distilled water were set as control (CK). Data show the means of three replicates ± standard deviation (SD). The different letters denote significant difference at *P* < 0.05 according to Turkey’s test

To further investigate the role of GA in HRW-mediated seed germination, the effect of HRW was studied after inhibiting GA biosynthesis with paclobutrazol (PAC, a GA biosynthesis inhibitor). As expected, compared to CK, GA treatment remarkably increased seed germination percentage (Fig. 3). For example, the seed germination percentage of GA treatment reached 59.03% at 66 h after imbibition, while that of CK was only 37.5%. It was observed that PAC treatment alone dramatically inhibited seed germination, since the seeds began to germinate at 66 h, and the germination percentage dropped to 53.5%, which was 68.8% lower than that of CK at 120 h (5 days) after imbibition (Fig. 3B). Notably, the promoted role of HRW in seed germination was strongly blocked after the inhibition of GA biosynthesis by PCA. Additionally, compared to HRW alone, HRW + PAC treatment decreased germination percentage at all measuring time points. Meanwhile, the seed germination percentages between PAC and HRW + PAC treatment had no significant difference. The average germination time (AGT) displayed the similar tendency (Fig. 3C). These results demonstrate that GA is essential for the promoting effect of HRW on seed germination in wax gourd.

### Role of ABA in HRW-mediated seed germination in wax gourd

**Fig. 4:**
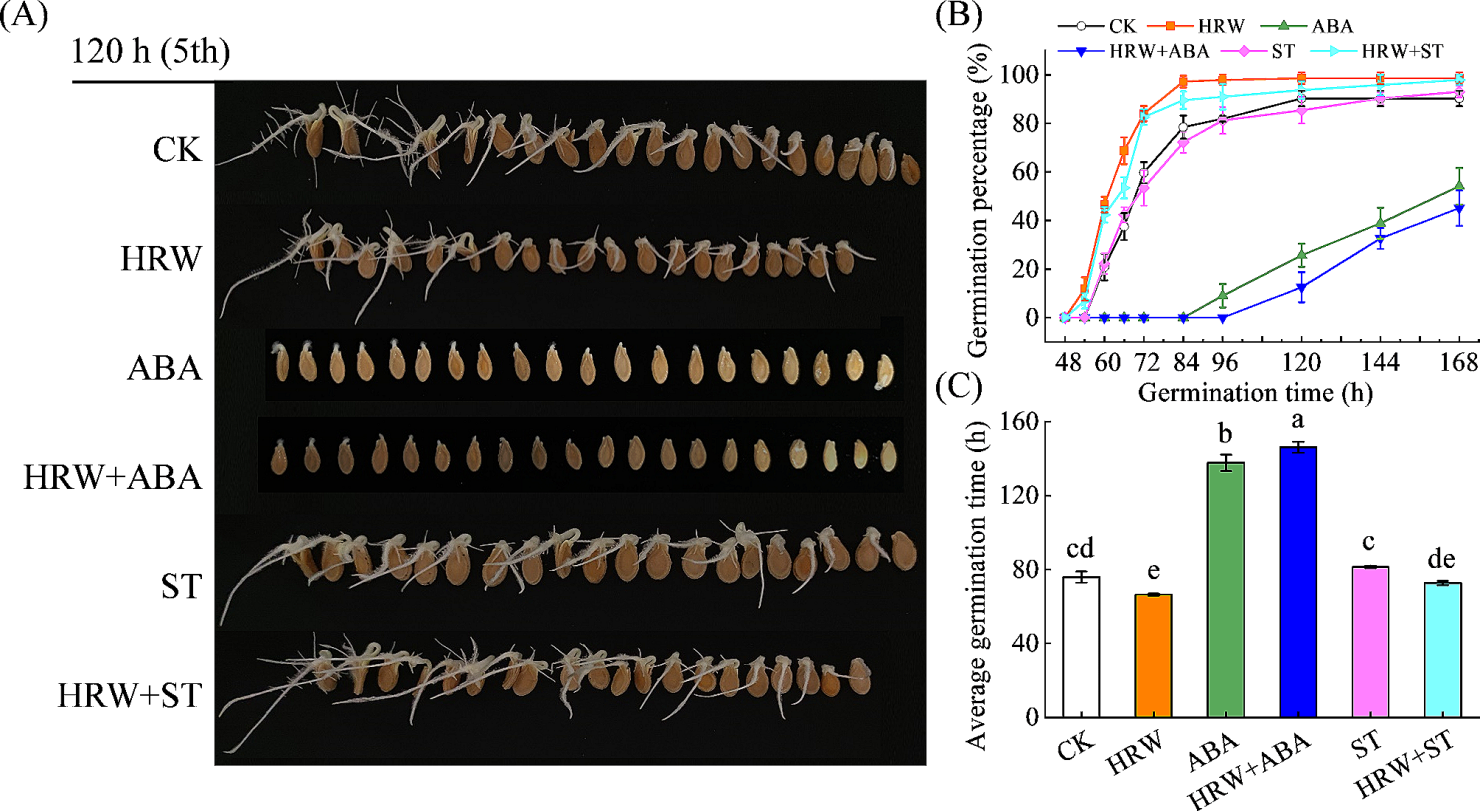
The role of ABA in HRW-mediated seed germination in wax gourd. Seeds were presoaked in distilled water (CK) or 0.15 mM ABA solution with or without 50% HRW (HRW). To inhibit ABA synthesis, seeds were pre-incubated in 0.3 mM sodium tungstate (ST) in the presence or absence of 50% HRW. After immersion for 6 hours, the seeds were transferred into an incubator at 30℃ for 168 hours (7 days). The photographs were taken at 120 hours (**A**). Meanwhile, seed germination percentage (**B**) and average germination time (**C**) were determined. Seeds immersed with distilled water were used as control (CK). The data are presented as mean ± standard deviation (SD). Means denoted with different letters indicate significant difference at *P* < 0.05 according to Turkey’s test

To investigate the function of ABA in HRW-promoted seed germination, the germination percentage were recorded after treatment with HRW, ABA, and sodium tungstate (ST, an ABA synthesis inhibitor). Consistent with previous studies, presoaking with exogenous ABA severely inhibited seed germination (Fig. 4). The seeds treated with ABA did not germinate until 96 h (4 days) after imbibition, while the CK and HRW treatments had reached to 81.9% and 97.9%, respectively. Notably, application of HRW did not alleviate the ABA-induced inhibition on seed germination in wax gourd. The germination percentage of ABA and HRW + ABA treatments were 54.2% and 45.1%, respectively, on the 168 h (7 days) after imbibition, which was significantly lower than that of the CK (Fig. 4B). The average germination time (AGT) of ABA and HRW + ABA were 137.8 and 146.1 h, which were 61.9 and 70.2 h later than that of CK (Fig. 4C). When ABA biosynthesis was inhibited by ST, the beneficial role of HRW in germination was restored. These findings indicate that ABA played a role downstream of HRW during seed germination and HRW could not overcome the inhibitory effect of ABA on seed germination.

### Transcriptome sequencing results and comparative analysis of differential transcripts

**Fig. 5:**
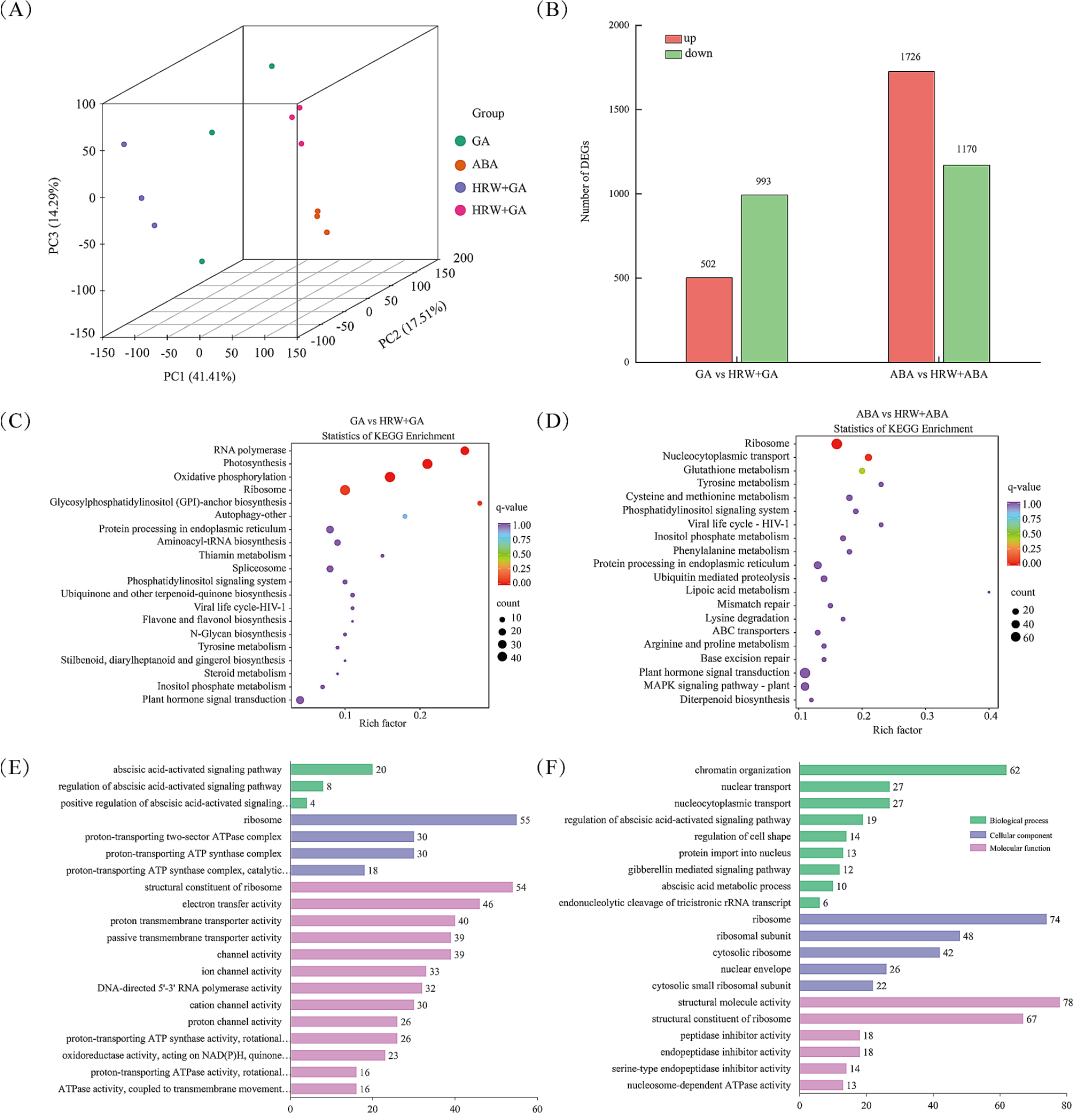
Analysis of transcriptome results based on mRNA-seq. Seeds were presoaked in GA_3_, or ABA solution with or without 50% HRW to form four treatments, namely GA, ABA, HRW+GA, and HRW+ABA. Seed samples were collected at 48 hours after imbibition for high-throughput mRNA sequencing, with three biological replicates for each treatment. (**A**) Number of DEGs (False Discovery Rate < 0.05, |log_2_Fold Change| ≥ 1) in two comparisons. (**B**) Venn diagram of DEGs. (**C**, **D**) KEGG metabolic pathway analysis of DEGs. The X and Y-axis represent enrichment factor and pathway names, respectively. A colored bubble represents q-value and bubble size indicates the number of DEGs. (**E**-**F**) GO terms of DEGs classified as biological, cellular, and molecular functions in GA vs HRW+GA and ABA vs HRW+ABA. The X and Y-axis represent DEGs numbers and pathway names, respectively

In this study, we performed transcriptomic sequencing of seeds treated with GA, ABA, HRW + GA, and HRW + ABA to explore the roles of GA and ABA metabolism on the HRW-mediated seed germination. A total of 84.12 G of clean bases was obtained, with each sample ranging from 6.26 to 7.95 G. As shown in Supplementary Table S2, the Q30 bases of 12 samples ranged from 92.6 to 94.97%, and the GC content ranged from 43.14 to 45.96%. Additionally, 560 million clean reads from 12 samples were reported by mapping unique sequences to the wax gourd genome, while 13.1 million reads were reported by multiple mapping (Supplementary Table S3). Three principal components of principal component analysis (PCA) score plot showed that the experiments were reproducible and reliable, with PC1, PC2, and PC3 accounting for 41.41%, 17.51%, and 14.29%, respectively (Fig. 5A). The differential groups for comparative study were created between GA vs. HRW + GA and ABA vs. HRW + ABA. A total of 4008 differentially expressed genes (DEGs) were identified, out of which 687 were unannotated genes. Specifically, there were 1495 DEGs (502 upregulated, 993 downregulated) in GA vs. HRW + GA and 2896 DEGs (1726 upregulated, 1170 downregulated) in ABA vs. HRW + ABA (Fig. 5B).

To facilitate the global analysis of DEGs regulated by HRW under GA or ABA treatment during seed germination, the Kyoto Encyclopedia of Genes and Genomes (KEGG) was used to identify the biological pathways of the DEGs and the Gene Ontology (GO) was carried out to classify the function categories of the DEGs [34, 35]. The KEGG pathways of DEGs in each comparison group for top 20 events were shown in Fig. 5C-D. Protein processing in endoplasmic reticulum (ko04141), phosphatidylinositol signaling system (ko04070), inositol phosphate metabolism (ko00562), ribosome (ko03010), and plant hormone signal transduction (ko04075) were the common pathways of DEGs in GA vs. HRW + GA and ABA vs. HRW + ABA. Additionally, we analyzed the top 50 GO enrichment in each comparison group, with the top 20 listed in Fig. 5E-F and the 21–50 listed in Supplementary Table S4 and Supplementary Table S5. In GA vs. HRW + GA, the GO functional annotation terms were enriched in cellular component and molecular function, accounting for 61.6% and 29.7%, respectively. These GO terms were mainly related to energy metabolism (proton-transporting ATP synthase complex, proton-transporting two-sector ATPase complex, NADH dehydrogenase activity), mitochondria (mitochondria membrane, mitochondria inner membrane, mitochondria envelope) and transporter or channel activity (inorganic cation transmembrane transporter activity, proton transmembrane transporter activity, ion channel activity, cation channel activity), which may provide sufficient energy for seed germination. The GO terms in ABA vs. HRW + ABA group mainly enriched in biological process. Several biological processes related to plant hormone signaling pathway were found in these two groups, such as abscisic acid-activated signaling pathway, regulation of abscisic acid-activated signaling pathway, response to gibberellin, gibberellin mediated signaling pathway (Fig. 5E and F).

### Expression profile of DEGs related to phytohormone signal transduction pathways

From the GO and KEGG analysis, we noticed that the phytohormone-signaling-related genes that involved in gibberellin, abscisic acid, and ethylene signaling pathways were different between GA vs. HRW + GA and ABA vs. HRW + ABA. To further clarify the mechanism of HRW-mediated seed germination from phytohormone signal transduction, we compared the transcript levels of phytohormone-signaling-related genes in GA, HRW + GA, ABA, and HRW + ABA seeds. Representative DEGs included *DELLA*, *phytochrome-interacting factor* (*PIF4*), *pyrabactin resistance*/*pyrabactin resistance1-like* (*PYR*/*PYL*), *type 2 C protein phosphatase* (*PP2C*), *SNF1-related protein kinase 2* (*SnRK2*), *abscisic acid-responsive element binding factors* (*ABFs*), *serine/threonine-protein kinase* (*CTR1*), *ethylene-insensitive protein 2* (*EIN2*), and *ethylene-responsive transcription factor 1*/*2* (*ERF1*/*2*).

**Fig. 6:**
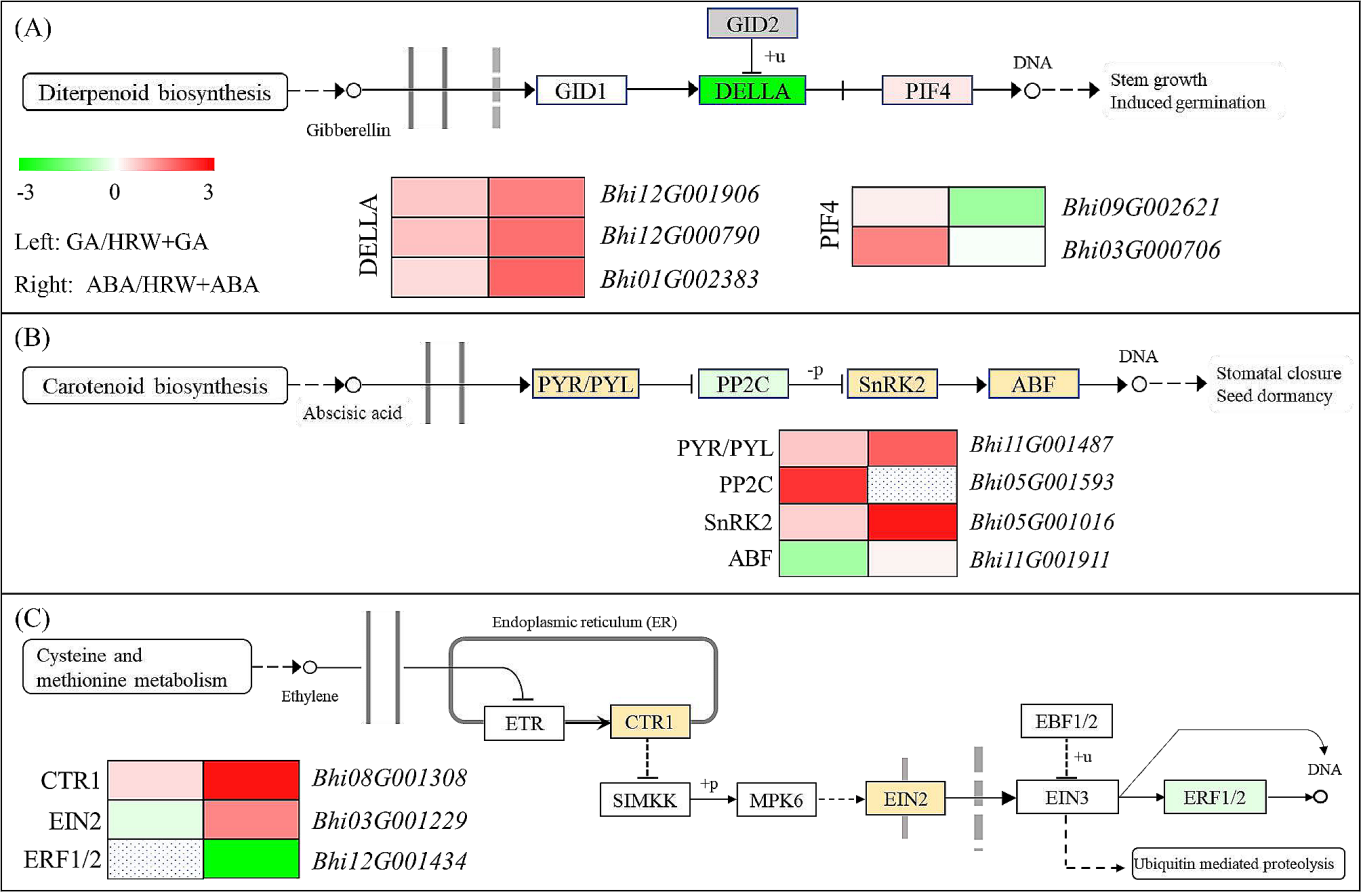
Transcription level analysis of genes associated to GA (**A**), ABA (**B**), and ethylene (**C**) signal pathways in GA vs HRW+GA and ABA vs HRW+ABA group. The Log_2_ Fold Change values corresponding to each gene in a group are used to generate heatmaps

In the GA signaling pathway, three *DELLA* genes (*Bhi01G002383*, *Bhi12G001906*, and *Bhi12G000790*) and two *PIF4* genes (*Bhi03G000706* and *Bhi09G002621*) were differently expressed between GA vs. HRW + GA and ABA vs. HRW + ABA groups (Fig. 6A). Compared with ABA alone, HRW + ABA treatment remarkably upregulated the expression of *DELLAs*, resulting in HRW’s failure to promote seed germination. In the presence of GA, HRW increased the expression of *PIF4* (*Bhi03G000706*), indicating that HRW played a role in promoting seed germination. However, in the presence of ABA, HRW downregulated or did not affect the expression of *PIF4* (*Bhi09G002621*). In the ABA signaling pathway, the transcript level of *PP2C* (*Bhi05G001593*) was upregulated in GA vs. HRW + GA group, and the transcript level of *ABF* was inhibited, indicating that HRW + GA treatment strongly restrained ABA signal transduction. Nevertheless, in the presence of ABA, HRW could not block ABA signal transduction pathway. For example, PP2C, as an important negative regulation factor, was no affected by HRW in the presence of ABA (Fig. 6B). Ethylene plays a positive role in seed germination. Results showed that some genes (*CTR1*, *EIN2*, *ERF1/2*) in the ethylene signaling pathway were induced by HRW in varying degrees when GA or ABA was present (Fig. 6C). The expression of these genes did not change in GA vs. HRW + GA. However, in the presence of ABA, HRW promoted the expression of ethylene negative regulator (*CTR1*) and inhibited the expression of ethylene positive regulator (*ERF1*/*2*).

### Expression analysis of genes related to inositol phosphate metabolism, GA, and ABA regulation process

**Fig. 7:**
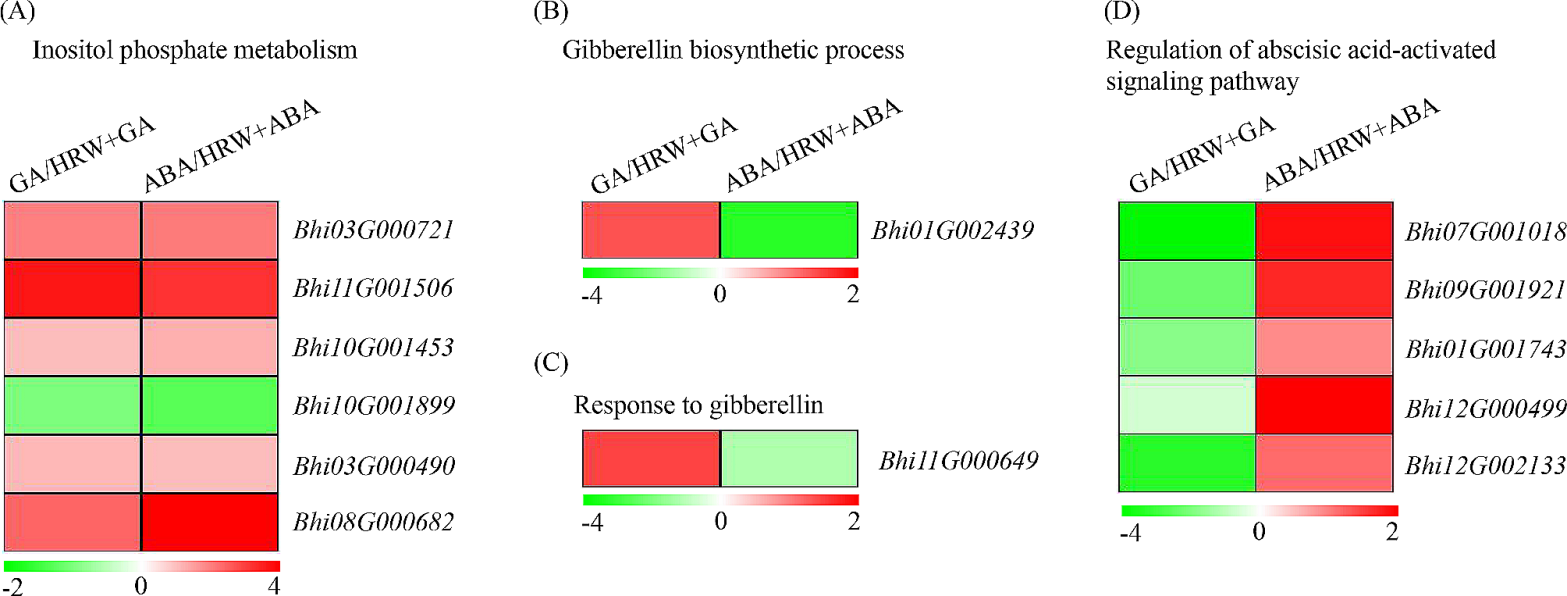
Transcription level analysis of genes associated to inositol phosphate metabolism (**A**), gibberellin biosynthetic process (**B**), response to gibberellin (**C**), and regulation of abscisic acid-activated signaling pathway (**D**) in GA vs HRW+GA and ABA vs HRW+ABA groups. The Log_2_ Fold Change values corresponding to each gene in a group are used to generate heatmaps

From the heat map in Fig. 7, it was found that several genes related to inositol phosphate metabolism, gibberellin biosynthetic process, response to gibberellin, and regulation of abscisic acid-activated signaling pathway differed in GA vs. HRW + GA and ABA vs. HRW + ABA groups. The description and classification of these genes were listed in Supplementary Table S6. For inositol phosphate metabolism, the six genes (*Bhi03G000721*, *Bhi11G001506*, *Bhi10G001453*, *Bhi10G001899*, *Bhi03G000490*, *Bhi08G000682*) showed the same expression pattern in GA vs. HRW + GA and ABA vs. HRW + ABA. Interestingly, HRW could enhance the expression of GA-related genes and inhibit the signal pathway activated by ABA. However, in the presence of ABA, HRW could not promote GA synthesis and response, nor could inhibit the signal pathway activated by ABA. These results may help to explain the reason for HRW-mediated seed germination from the perspective of signal regulation.

### Verification of transcriptome data by quantitative real-time PCR

**Fig. 8:**
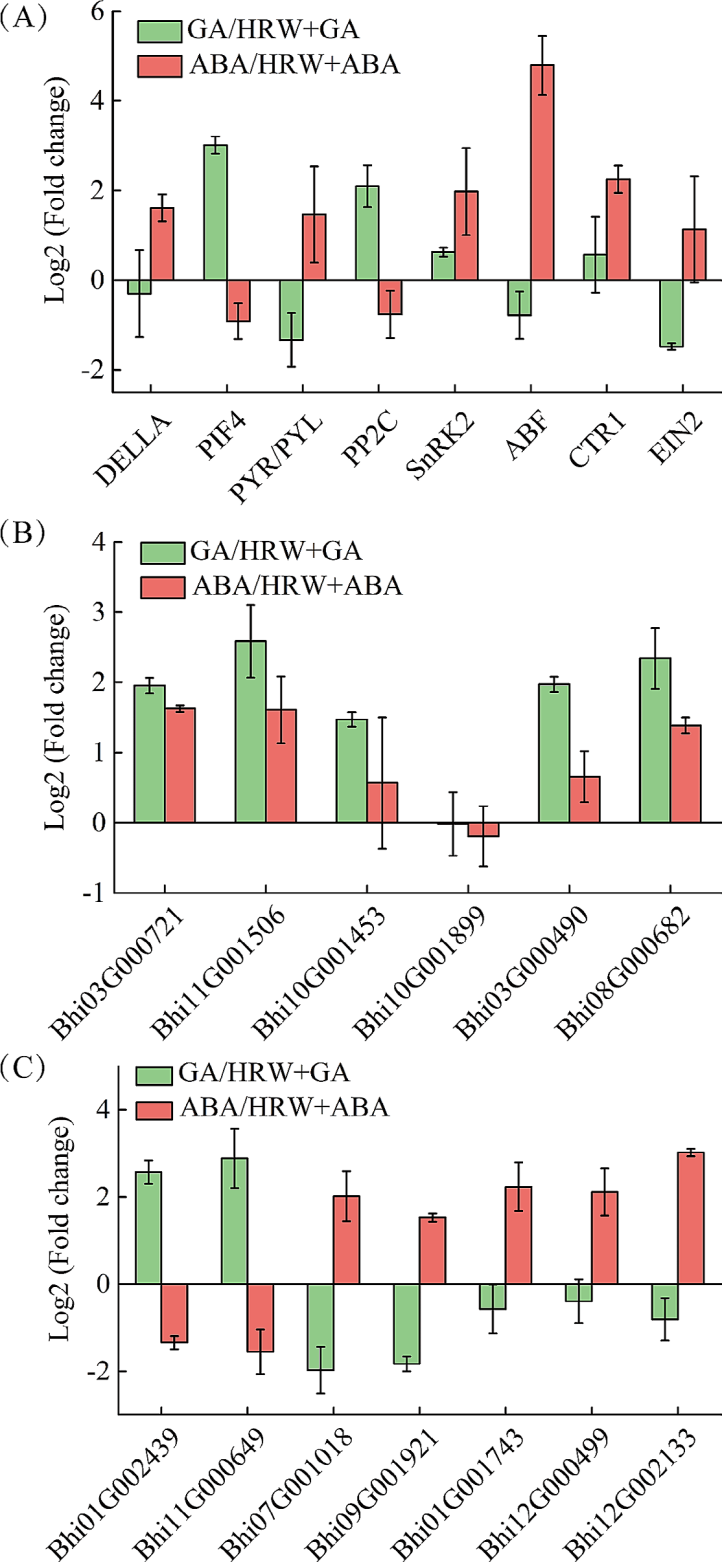
Expression analysis of DEGs via qRT-PCR in GA vs. HRW + GA and ABA vs. HRW + ABA group. (**A**) Expression analysis of genes associated to GA, ABA, and ethylene signal pathways. (**B**) Expression analysis of genes associated to inositol phosphate metabolism. (**C**) Expression analysis of genes associated to gibberellin biosynthetic process (*Bhi01G002439*), response to gibberellin (*Bhi11G000649*), and regulation of abscisic acid-activated signaling pathway. The wax gourd *BhiUBCP* (*Bhi07G001302*) gene was set as an internal control. Data of figure are the means of Log_2_ Fold Change values corresponding to each gene in a group. GA, gibberellin; ABA, abscisic acid

To ensure the reliability of our transcriptome dataset, a quantitative real-time PCR (qRT-PCR) experiment was conducted using DEGs associated with various pathways, including GA-, ABA-, and ethylene-related pathway, and inositol phosphate metabolism. To ensure the reliability of the qRT-PCR results, we employed two internal reference genes for normalization. The results using *BhiUBCP* as the internal reference were shown in Fig. 8, while the results using *BhiUBQ* as the internal reference gene were presented in Supplementary Figure S1. The qRT-PCR analysis results demonstrated a consistent pattern of transcript level changes with those observed in the transcriptome sequencing analysis. This agreement between the two independent methods further supported the conclusions drawn from the transcriptome data and strengthened the reliability of findings.

## Discussion

Seed germination is a critical stage in the life cycle of higher plants. As an important gaseous regulator, H_2_ was discovered to promote seed germination as far back as 1964 [31]. More recently, several studies have implicated the positive participation of H_2_ in seed germination, particularly under stress conditions such as drought, salt, and heavy metals [27, 32, 33, 36]. Consistent with previous observations, we found that both 50% and 75% HRW treatment could increase seed germination percentage in wax gourd under stress-free condition (Fig. 1). Considering the lack of difference between the germination percentage of 50% and 75% HRW, we believe that a 50% HRW concentration is appropriate concentration for promoting seed germination in wax gourd. Due to safe and easily available, HRW might be a good option to promote seed germination in future agriculture.

In higher plants, it is widely known that GA and ABA play major determinants in regulating seed germination and dormancy in an antagonistic manner [6]. Xu et al. found that H_2_ pretreatment significantly increased GA contents while reducing ABA contents, thereby restoring GA/ABA balance, in aluminum-stressed rice seeds [33]. Consequently, we investigated the effects of HRW pretreatment on GA and ABA contents and the expression of some biosynthetic and metabolism genes (Fig. 2). Related experiments illustrated that HRW pretreatment significantly increased GA contents by upregulating GA biosynthetic genes (*BhiGA3ox*, *BhiGA2ox*, and *BhiKAO*). Moreover, the promotive effects of HRW on seed germination could be reversed by PAC, a GA biosynthesis inhibitor (Fig. 3), which is consistent with the work by Xu et al. [33]. Unlike the reduction of H_2_ on ABA content in rice seeds under Al stress [33], the current study observed that HRW treatment did not significantly change ABA content (Fig. 2). Meanwhile, the expression of ABA biosynthetic gene (*BhiNCED6*) and degradation gene (*BhiCYP707A2*) remained unchanged significantly in HRW pretreated seeds, while the transcript level of ABA receptor gene (*BhiPYL*) was downregulated (Fig. 2). Interestingly, the inhibition of ABA on seed germination cannot be alleviated by HRW (Fig. 4). Based on these results, we inferred that GA and ABA might be the necessary downstream targets for H_2_ to regulate germination of wax gourd seed.

Generally, seed germination is tightly regulated by various signaling pathways, with key involvement of phytohormones such as ABA and GA [37]. Understanding the roles of H_2_ in regulating GA and ABA during seed germination and dormancy is crucial for comprehending H_2_ signaling in plants. Based on the transcriptome sequencing analysis, we apprehended the signal mechanisms underlying HRW-mediated seed germination by GA and ABA signaling pathway. During seed germination, a large number of regulatory factors related with GA and ABA signaling pathway were changed in transcriptome level [13]. In this present study, the KEGG enrichment analysis revealed that plant hormone signal transduction is the most important enrichment pathway in GA vs. GA + HRW and ABA vs. ABA + HRW groups (Fig. 5). Additionally, the process of seed germination also requires energy because it involves various biochemical and physiological changes that enable the dormant seed to resume growth. The starch, protein, and lipids stored in seeds are broken down into simple sugars, amino acids, and fatty acids, providing energy sources for cell division, elongation, and other growth processes [38–40]. We found that a set of DEGs in GA vs. HRW + GA group were enriched in energy metabolism-related processes according to GO terms. Seed germination requires energy, and H_2_ has been reported to increase soluble sugar content, thereby supplying energy for seed germination [32, 36]. Further research can be performed to investigate the more detailed mechanism of H_2_-mediated seed germination by regulating ABA and GA signaling from the perspective of sugar and energy metabolism. Moreover, inositol phosphate metabolism is a common enrichment pathway of GA vs. HRW + GA and ABA vs. HRW + ABA, which is a key metabolic pathway to provide energy and raw materials for seed germination [41]. The fact that there was no difference in the transcription levels of DEGs in this pathway between two groups (Figs. 7 and 8), indicating that inositol phosphate metabolism pathway might be upstream of GA and ABA or not affected by HRW.

Seed germination is mainly achieved through the regulation of GA and ABA signaling. With respect to GA signaling, GID1 (GA receptor) and DELLA (negative regulator) are the main components involved in early perception and signaling [42–44]. In the presence of GA, its receptors GID1 interacts with DELLA and form a ubiquitination complex via interaction with GID2, leading to the degradation of DELLAs [13, 17, 43, 45]. In the current study, HRW inhibits the expression of DELLA to ensure the transmission of GA signals, leading to the promotion of seed germination (Fig. 8). Furthermore, there are three GA-related pathway genes (*Bhi03G000706*, *Bhi01G002439*, and *Bhi11G000649*) positively regulated by HRW (Figs. 6 and 8). Similarly, in the case of ABA signaling, there are three main components involved in early perception and signaling: ABA receptor (PYR/PYL) proteins and SnRK2 proteins as positive regulators, and PP2C proteins as negative regulators. In the ABA signaling pathway, PYR/PYLs can bind PP2Cs and form a stable complex, leading to phosphorylation and activation of SnRK2s [46, 47]. In the presence of GA, HRW has the ability to attenuate the ABA signaling pathway; however, when ABA is present, HRW does not exhibit inhibitory effects on the ABA signaling pathway (Figs. 6 and 8). Ethylene, as a plant hormone, also plays a role in seed germination [48]. Ethylene response factors (ERFs) are key transcription factors that play a regulatory role downstream of the ethylene signal. It establishes connections between ethylene, GA and ABA signals [49, 50]. The results of this study showed that HRW had difference effects on *CTR1*, *EIN2*, and *ERF1/2* expression in the ethylene signaling pathway when GA or ABA existed, suggesting that HRW mediated ethylene signaling pathway by regulating the balance between GA and ABA (Figs. 6 and 8). Together, it appears that the regulation of H_2_ on wax gourd seed germination is mainly achieved through plant hormone signal transduction.

## Conclusion

**Fig. 9:**
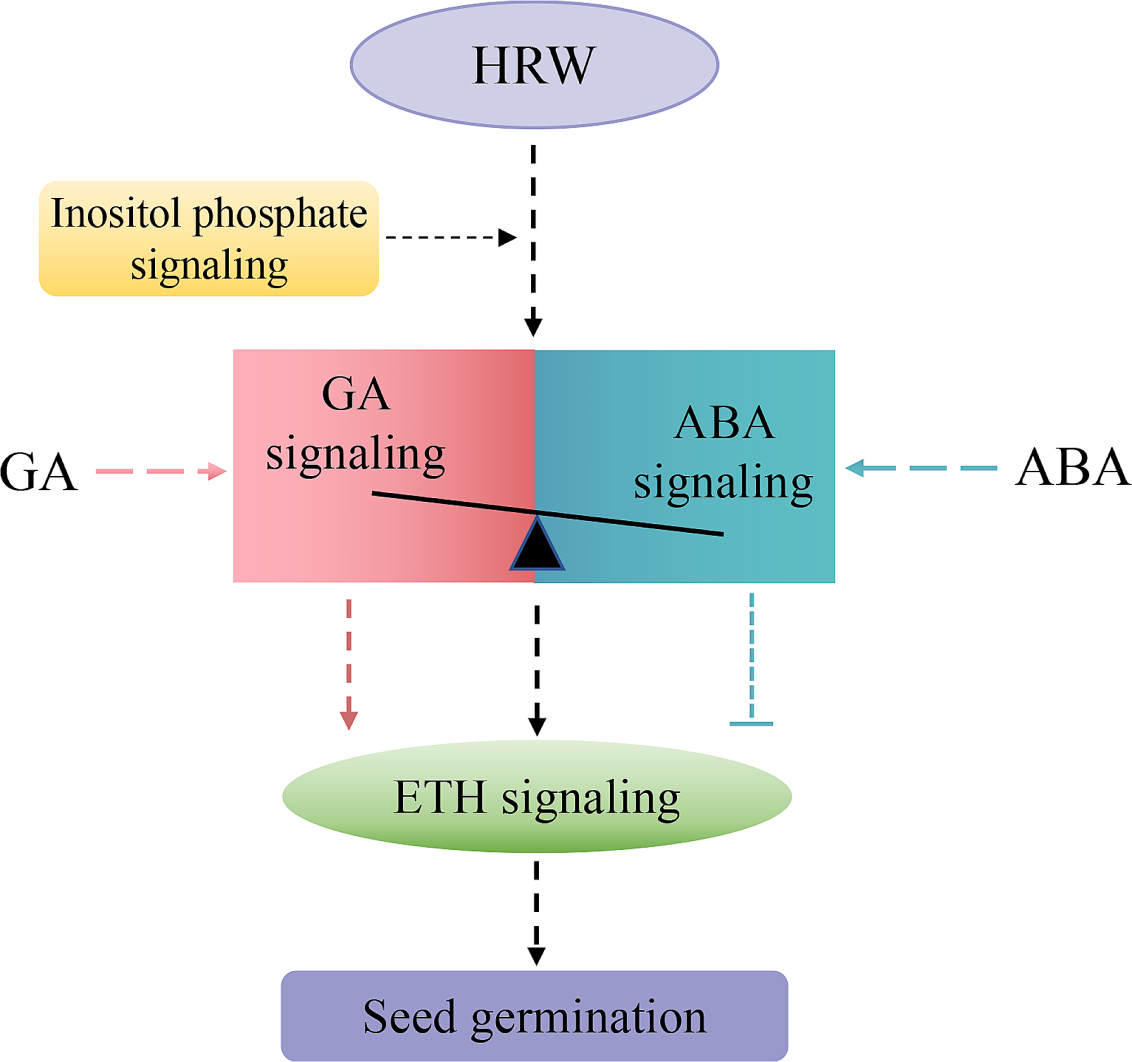
The possible action pattern of HRW regulating seed germination. HRW regulates the balance between GA and ABA signals through inositol phosphate signaling to mediate seed germination via the downstream ethylene signaling. When the GA content is high in seeds, HRW can activate ethylene pathway through GA signal to promote seed germination. Contrary, for seeds with high ABA content (such as year-old seeds or dormant seeds), ABA can block the connection between HRW and ethylene signal, resulting in inhibition of seed germination. GA, gibberellin; ABA, abscisic acid; ETH, ethylene

In conclusion, this study demonstrated that HRW treatment promoted seed germination in a dose-dependent manner, with 50% HRW being the optimal concentration (Fig. 1). Treatment with 50% HRW significantly increased GA content and maintained ABA content, resulting in an increase in GA/ABA ratio. In addition, HRW could not induce seed germination after inhibiting GA synthesis or increasing ABA content. Analysis of high-throughput mRNA sequencing revealed that, in the presence of GA or ABA, HRW enhanced the positive roles of GA but did not alleviate the inhibitory effects of ABA during seed germination, which mainly achieved by regulating plant hormone signaling, including GA, ABA, and ethylene signal transduction (Fig. 9). These findings suggested that HRW might not promote germination in seeds with high ABA content, such as year-old seeds. This result may provide a basis for the application of HRW on seed germination to avoid negative effects.

## Materials and methods

### Preparation of hydrogen-rich water (HRW)

Purified hydrogen gas (H_2_, 99.99%, v/v) generated from a H_2_ generator (HIM-22, CAWOLO, Foshan, China) was bubbled into 1000 mL of distilled water at a rate of 150 mL min^− 1^ for 30 min at 25°C, to obtain a saturated solution of H_2_ [26]. The concentration of H_2_ in saturated HRW is 1.260 mg L^− 1^. Then, the saturated HRW was immediately diluted with distilled water to the required concentrations (25%, 50%, 75%, and 100%, v/v). The H_2_ concentrations in 25%, 50%, 75%, and 100% HRW are 0.315, 0.630, 0.945, and 1.260 mg L^− 1^, respectively. H_2_ concentration in freshly prepared HRW was determined using a hydrogen portable meter (Trustlex Co., Ltd., ENH-1000, Yokohama, Japan) according to Zeng and Yu [51].

### Plant materials, experimental design, and germination conditions

Wax gourd (*Benincasa hispida* L. cv. Tiezhu2) seeds used in this study were supplied by Vegetable Research Institute of Guangdong Academy of Agricultural Sciences, Guangdong Province, China. The seeds were immersed in different concentrations of HRW for 6 h, and then placed in Petri dishes (12 × 12 cm) containing three filter papers moistened with distilled water. Then, the seeds were incubated at the temperature of 30°C for 120 h (5 d) in a germinator in the dark. A seed whose radicle breaks through the seed coat was considered to germinate. The number of germinated seeds was counted at a specific time, which is the number of germinated seeds in that period. The average germination time (AGT) was calculated to reflect the uniformity and consistency of seed germination during the whole process. The AGT is calculated as follow.

AGT=∑(f×X)/∑X. Where, x refers to the numbers of new germinated seeds at f hour, f refers to the germination time, and ∑X is the total number of germinated seeds.

Each treatment was carried out on three duplications with 48 seeds in each Petri dish. The seeds were sampled at 48 h after imbibition from each treatment, rapidly frozen in liquid nitrogen, and stored at − 80°C until analysis.

### Chemical treatments

To investigate the mechanisms by which H_2_ balances GA and ABA to regulate seed germination, wax gourd seeds were presoaked in different solutions including HRW, gibberellic acid (GA_3_, Sigma-Aldrich), abscisic acid (ABA, Sigma-Aldrich), paclobutrazol (PAC, a GA synthesis inhibitor, Sigma-Aldrich), sodium tungstate (ST, an ABA synthesis inhibitor, Sigma-Aldrich), or distilled water for 6 h. Test solutions used for treating wax gourd seeds were as follows: HRW (50%, v/v), GA_3_ (0.9 mM), ABA (0.15 mM), PAC (0.3 mM), ST (0.3 mM), mixture of HRW and GA_3_, ABA, PAC, or ST. After rinsing with distilled water, wax gourd seeds were incubated for 168 h (7 d) under 30°C as described in the previous.

### Quantification of endogenous ABA and GA

Fresh seed samples were collected, immediately frozen in liquid nitrogen, ground into powder (30 Hz, 1 min), and stored at − 80°C for measuring ABA and GA contents. The extraction and quantification of ABA and GA were performed as described previously [52]. Briefly, 0.5 g of seed tissues were weighed into 10 mL centrifuge tubes and dissolved in 5 mL methanol/water/formic acid (15:4:1, v/v/v) to extract ABA and GA by overnight incubation. The mixture was vortexed and centrifugated for 5 min (12,000 *g*, 4°C). The supernatant was transferred to clean microtubes, followed by evaporation to dryness and dissolved in 100 µL 80% methanol (v/v), and filtered through a 0.22 μm membrane filter for further LC-MS/MS analysis. Then, the hormone contents of ABA and GA were detected by MetWare (http://www.metware.cn/) based on the AB Sciex QTRAP 6500 LC-MS/MS platform.

### RNA isolation and qRT-PCR analysis

Total RNA from seeds were extracted using an RNA isolation kit (TIANGEN, Beijing, China). After removing residual DNA from the total RNA, the first-strand cDNA was synthesized using the FastKing gDNA Dispelling RT SuperMix (TIANGEN, Beijing, China) according to the manufacturer’s instructions. The gene-specific primers were designed based on mRNA sequences using Primer Premier 6 (Supplementary Table S1 and Supplementary Table S7). The qRT-PCR was performed with a Bio-Rad CFX Connect Real-Time PCR Detection System using Talent qPCR PreMix (TIANGEN, Beijing, China). The amplification program of PCR was run at 95°C for 3 min, followed by 40 cycles of 5 s at 95°C and 15 s at 60°C. The wax gourd *BhiUBCP* (*Bhi07G001302*) and *BhiUBQ* (*Bhi10G000739*) were set as internal controls. The quantification of mRNA levels was performed as described previously [53].

### Transcriptome sequencing and differential expression analysis

Twelve independent mRNA libraries from seed samples for four treatments (GA, ABA, HRW + GA, and HRW + ABA), with three biological replicates for each treatment, were generated using NEBNext^®^UltraTM RNA Library Prep Kit for Illumina^®^ (NEB, USA) following manufacturer’s recommendations. Library quality was assessed on the Agilent Bioanalyzer 2100 system. The library construction and sequencing of mRNA were performed on the Illumina sequencing platform by Metware Biotechnology Co., Ltd. (Wuhan, China). After removal of reads containing adapter, poly-N and low-quality bases, clean reads were mapped to wax gourd reference genome (http://cucurbitgenomics.org/v2/). The expression level of each gene was calculated and normalized to generate FPKM (fragments per kilobase of exon per million mapped fragments) based on feature counts [54]. The differential expression analysis between two groups was using DESeq2 (v1.22.1) software [55]. The false discovery rate (FDR) was used to determine the threshold of the *P*-value in multiple tests [56]. The FDR < 0.05 and |log_2_foldchange| ≥ 1 was set as the threshold for significant difference expression.

### Statistical analysis

The experimental layout was a completely randomized design with three independent biological replicates. Data are the means ± SE from three replicates. The statistical analysis was performed using the Student’s *t*-test and one-way analysis of variance (ANOVA) followed by Turkey’s test. A value of *P* < 0.05 was considered significant differences between treatment means.

### Electronic supplementary material

Below is the link to the electronic supplementary material.

Supplementary Material 1

## Data Availability

The datasets generated and analyzed are available in the NCBI Sequence Read Archive under the following accessions: PRJNA900522. Other data are available in the supplementary material.

## Abbreviations

HRW: Hydrogen-rich water
GA: Gibberellin
ABA: Abscisic acid
AGT: Average germination time
PAC: Paclobutrazol
ST: Sodium tungstate
