# Supplementary material for: The GA and ABA signaling is required for hydrogen-mediated seed germination in wax gourd
Source: BMC Plant Biol. 2024 Jun 13;24:542. doi: 10.1186/s12870-024-05193-3 (PMC11177465; doi:10.1186/s12870-024-05193-3)
Supplement: Supplementary file 1 — Supplementary Material 1 [file 12870_2024_5193_MOESM1_ESM.docx]

**Supplementary Information**

Supplemental Table 1 Primers used for quantitative real-time PCR analysis in Figure 2.

Supplemental Table 2 Sequencing data Statistics.

Supplemental Table 3 Statistics on data mapping.

Supplemental Table 4 The top 21-50 GO enrichment in GA vs HRW+GA group.

Supplemental Table 5 The top 21-50 GO enrichment in ABA vs HRW+ABA group.

Supplemental Table 6 Description and classification of genes in Figure 7.

Supplemental Table 7 Quantitative real-time PCR primers used for verifying transcriptome data.

Supplemental Figure 1 Expression analysis of DEGs via qRT-PCR in GA vs HRW+GA and ABA vs HRW+ABA group.

Supplemental Table 1 Primers used for quantitative real-time PCR analysis in Figure 2

| Gene ID | Gene name | Forward sequence (5’-3’) | Reverse sequence (5’-3’) | Description |
| --- | --- | --- | --- | --- |
| *Bhi07G001302* | *BhiUBCP* | ACCGACAGTTCGCTTTGTGTCT | GGGTCACACAGCAAGGACTGAA | Internal reference |
| *Bhi10G000739* | *BhiUBQ* | CCTAACTGGGAAGACGAT | CAAGACCAAGTGAAGGGT |  |
| *Bhi12G001328* | *BhiNCED6* | AGTAATCACTCCGCCACCTCCA | TAGGGACGCCGCCAAATTCTGA | 9-cis-epoxycarotenoid dioxygenase NCED6 |
| *Bhi09G002591* | *BhiPYL* | GCCCTTTGTTCGCAGCTTTGAT | AGACCACCGTCACCTCTCTGAT | Abscisic acid receptor PYL |
| *Bhi08G000494* | *BhiCYP707A2* | ATTCGAGGTGCCACCTAGACCA | TAGCCATCTCACTGCCTGGACA | Abscisic acid 8’-hydroxylase |
| *Bhi09G000068* | *BhiGA3ox* | AAGTCATTAACCACGGCGTCCC | TGAAGCCTTCGGACCACATACG | Gibberellin 3-beta-dioxygenase |
| *Bhi08G001697* | *BhiGA2ox* | CGGCGATGTGGGTTGGATTGAA | CAAGCCATCTCCTTCACAGCCA | Gibberellin 2-beta-dioxygenase |
| *BhiUN298G4* | *BhiKAO* | TGGCTATGGCTGATCGGAGCTT | AGATTGTAACGACGGCGGTGAC | Ent-kaurenoic acid oxidase |

Supplemental Table 2 Sequencing data Statistics

| Samples | Raw Reads | Clean Reads | Clean Base (G) | Error Rate (%) | Q20 (%) | Q30 (%) | GC Content (%) |
| --- | --- | --- | --- | --- | --- | --- | --- |
| GA-1 | 47441610 | 46652524 | 7 | 0.03 | 97.55 | 93.15 | 43.48 |
| GA-2 | 43086376 | 41726054 | 6.26 | 0.03 | 97.5 | 93.03 | 43.99 |
| GA-3 | 50667152 | 48259238 | 7.24 | 0.03 | 97.28 | 92.6 | 43.14 |
| ABA-1 | 50669738 | 49711052 | 7.46 | 0.03 | 97.27 | 92.7 | 44.17 |
| ABA-2 | 43999272 | 43164896 | 6.47 | 0.03 | 97.41 | 92.97 | 44.58 |
| ABA-3 | 46020306 | 44972302 | 6.75 | 0.03 | 97.34 | 93.07 | 43.57 |
| HRW+GA-1 | 51690704 | 50844714 | 7.63 | 0.03 | 97.65 | 93.41 | 43.73 |
| HRW+GA-2 | 47989700 | 47021912 | 7.05 | 0.03 | 97.61 | 93.26 | 43.64 |
| HRW+GA-3 | 45014116 | 44337668 | 6.65 | 0.03 | 97.92 | 93.97 | 44.18 |
| HRW+ABA-1 | 44514442 | 43677710 | 6.55 | 0.03 | 97.55 | 93.17 | 45.63 |
| HRW+ABA-2 | 53774592 | 52987954 | 7.95 | 0.03 | 97.58 | 93.23 | 45.96 |
| HRW+ABA-3 | 48172938 | 47406294 | 7.11 | 0.03 | 97.63 | 93.32 | 45.05 |

Supplemental Table 3 Statistics on data mapping

| Samples | Total Reads | Reads mapped | Unique mapped | Multi mapped |
| --- | --- | --- | --- | --- |
| GA-1 | 46652524 | 43001429 (92.17%) | 41937675 (89.89%) | 1063754 (2.28%) |
| GA-2 | 41726054 | 38939363 (93.32%) | 37998268 (91.07%) | 941095 (2.26%) |
| GA-3 | 48259238 | 43542090 (90.23%) | 42521040 (88.11%) | 1021050 (2.12%) |
| ABA-1 | 49711052 | 41753536 (83.99%) | 40990559 (82.46%) | 762977 (1.53%) |
| ABA-2 | 43164896 | 39934729 (92.52%) | 39178950 (90.77%) | 755779 (1.75%) |
| ABA-3 | 44972302 | 41280174 (91.79%) | 40559420 (90.19%) | 720754 (1.60%) |
| HRW+GA-1 | 50844714 | 48827598 (96.03%) | 47502285 (93.43%) | 1325313 (2.61%) |
| HRW+GA-2 | 47021912 | 45237335 (96.20%) | 43967010 (93.50%) | 1270325 (2.70%) |
| HRW+GA-3 | 44337668 | 42698935 (96.30%) | 41503295 (93.61%) | 1195640 (2.70%) |
| HRW+ABA-1 | 43677710 | 40177449 (91.99%) | 38881906 (89.02%) | 1295543 (2.97%) |
| HRW+ABA-2 | 52987954 | 48641182 (91.80%) | 46731850 (88.19%) | 1909332 (3.60%) |
| HRW+ABA-3 | 47406294 | 44551372 (93.98%) | 43711672 (92.21%) | 839700 (1.77%) |

Supplemental Table 4 The top 21-50 GO enrichment in GA vs HRW+GA group

| Ontology | Description | Gene count |
| --- | --- | --- |
| Molecular function | heme transmembrane transporter activity | 6 |
| Molecular function | electron transporter, transferring electrons within the cyclic electron transport pathway of photosynthesis activity | 10 |
| Molecular function | NADH dehydrogenase (ubiquinone) activity | 16 |
| Molecular function | ATPase-coupled cation transmembrane transporter activity | 17 |
| Molecular function | ATPase-coupled ion transmembrane transporter activity | 17 |
| Molecular function | NADH dehydrogenase activity | 17 |
| Molecular function | NADH dehydrogenase (quinone) activity | 17 |
| Molecular function | NAD(P)H dehydrogenase (quinone) activity | 17 |
| Molecular function | chlorophyll binding | 20 |
| Molecular function | oxidoreductase activity, acting on NAD(P)H | 24 |
| Molecular function | rRNA binding | 34 |
| Molecular function | 5'-3' RNA polymerase activity | 37 |
| Molecular function | RNA polymerase activity | 37 |
| Molecular function | ligase activity | 38 |
| Molecular function | inorganic cation transmembrane transporter activity | 52 |
| Molecular function | structural molecule activity | 54 |
| Cellular component | proton-transporting ATP synthase complex, coupling factor F(o) | 12 |
| Cellular component | proton-transporting two-sector ATPase complex, proton-transporting domain | 12 |
| Cellular component | small ribosomal subunit | 17 |
| Cellular component | proton-transporting two-sector ATPase complex, catalytic domain | 18 |
| Cellular component | photosystem | 21 |
| Cellular component | respirasome | 25 |
| Cellular component | mitochondrial inner membrane | 34 |
| Cellular component | mitochondrial membrane | 49 |
| Cellular component | mitochondrial envelope | 49 |
| Cellular component | ribosome | 55 |
| Biological process | mitochondrial translation | 9 |
| Biological process | glycerolipid biosynthetic process | 17 |
| Biological process | protein-chromophore linkage | 19 |
| Biological process | photosynthesis | 41 |

Supplemental Table 5 The top 21-50 GO enrichment in ABA vs HRW+ABA group

| Ontology | Description | Gene count |
| --- | --- | --- |
| Molecular function | 1,3-beta-D-glucan synthase activity | 7 |
| Molecular function | endopeptidase regulator activity | 18 |
| Molecular function | peptidase regulator activity | 18 |
| Molecular function | protein self-association | 27 |
| Cellular component | 1,3-beta-D-glucan synthase complex | 7 |
| Cellular component | RNA polymerase III complex | 8 |
| Cellular component | mediator complex | 13 |
| Cellular component | polysomal ribosome | 15 |
| Cellular component | polysome | 17 |
| Cellular component | small ribosomal subunit | 26 |
| Biological process | endonucleolytic cleavage involved in rRNA processing | 6 |
| Biological process | (1->3)-beta-D-glucan biosynthetic process | 7 |
| Biological process | maturation of 5.8S rRNA from tricistronic rRNA transcript (SSU-rRNA, 5.8S rRNA, LSU-rRNA) | 10 |
| Biological process | nucleosome organization | 15 |
| Biological process | ribonucleoprotein complex localization | 15 |
| Biological process | ribonucleoprotein complex export from nucleus | 15 |
| Biological process | endosomal transport | 16 |
| Biological process | protein-containing complex localization | 16 |
| Biological process | RNA export from nucleus | 18 |
| Biological process | protein export from nucleus | 19 |
| Biological process | response to gibberellin | 21 |
| Biological process | photomorphogenesis | 21 |
| Biological process | nuclear export | 22 |
| Biological process | RNA localization | 24 |
| Biological process | nucleocytoplasmic transport | 27 |
| Biological process | response to red or far-red light | 35 |
| Biological process | gene silencing | 36 |
| Biological process | regulation of cellular component organization | 62 |
| Biological process | intracellular protein transport | 72 |
| Biological process | regulation of growth | 63 |
| Biological process | intracellular protein transport | 72 |

Supplemental Table 6 Description and classification of genes in Figure 7

| Gene ID | Description | GO classification |
| --- | --- | --- |
| *Bhi03G000721* | phosphatidylinositol-3,4,5-trisphosphate 3-phosphatase and dual-specificity protein phosphatase PTEN | Phosphatidylinositol signaling system |
| *Bhi11G001506* | phosphatidylinositol 4-phosphatase |  |
| *Bhi10G001453* | CDP-diacylglycerol--inositol 3-phosphatidyltransferase |  |
| *Bhi10G001899* | inositol-1,3,4-trisphosphate 5/6-kinase / inositol-tetrakisphosphate 1-kinase |  |
| *Bhi03G000490* | phosphatidate cytidylyltransferase |  |
| *Bhi08G000682* | phosphatidate cytidylyltransferase |  |
| *Bhi01G002439* | gibberellin 2beta-dioxygenase | Gibberellin biosynthetic process |
| *Bhi11G000649* | Phytohormone-binding protein | Response to gibberellin |
| *Bhi07G001018* | E3 ubiquitin-protein ligase KEG | Regulation of abscisic acid-activated signaling pathway |
| *Bhi09G001921* | E3 ubiquitin-protein ligase KEG |  |
| *Bhi01G001743* | Receptor-like protein kinase FERONIA |  |
| *Bhi12G000499* | dual specificity protein kinase YAK1 |  |
| *Bhi12G002133* | RNA polymerase II C-terminal domain phosphatase-like 3/4 |  |

Supplemental Table 7 Quantitative real-time PCR primers used for verifying transcriptome data

| Gene ID | Forward sequence (5’-3’) | Reverse sequence (5’-3’) | Description |
| --- | --- | --- | --- |
| *Bhi12G001906* | TGCTATGTGGGTGCTGCTGAAC | GCCGCCGCATTTGTCTAAAGGA | DELLA |
| *Bhi09G002621* | CACGAGGCAGCAATGGAACTGT | CTGCGTGGATGTGGTTGGCATA | Phytochrome-interacting factor (PIF4) |
| *Bhi05G001593* | GTCGCAAGGGCATAAACCAGGA | ACCGTAAGGTCCATGTCCATCG | Type 2C protein phosphatase (PP2C) |
| *Bhi11G001487* | CCGCCTCAATCCATTGCCAGAA | GGCAGCTCTTGACGAAGTGCTT | Pyrabactin resistance/pyrabactin resistance1-like (PYR/PYL) |
| *Bhi05G001016* | ACTGGATGGAAGTCCTGCTCCT | CATGACCACACGTCCGCAATCT | SNF1-related protein kinase 2 (SnRK2) |
| *Bhi11G001911* | TTGCCACCGCCTCAGCAATATG | ACCACCGTTCACCACTCTTCCA | Abscisic acid-responsive element binding factors (ABF) |
| *Bhi08G001308* | ACAGGAGCCACATTCCGTTCCA | AGGTGCCATCCAGCGGTAAGTT | Serine/threonine-protein kinase (CTR1) |
| *Bhi03G001229* | AGCAGGTGTTTAGGAGCCCAGT | GCACAATGGAGCCAACCAGGAA | Ethylene-insensitive protein 2 (EIN2) |
| *Bhi03G000721* | TGCAGTCAACAATTCGGCTCCA | GCTGCTTCAGGTGGTTGTGGAT | Inositol phosphate metabolism |
| *Bhi11G001506* | CTCCCTTGTGATGCGGCTTTGA | TGGTCTTGCCGTCCATCCTTCA |  |
| *Bhi10G001453* | ACTGTTGTGTGTCATGCGAGGT | TCTGCCGCCGTCTTCATCTGTA |  |
| *Bhi10G001899* | TGTGGTGAGCCAGGTGAAGGTT | TGAACATCTCTCCGCCGTGGTT |  |
| *Bhi03G000490* | GCTTCACTGTGATCCTGGTCCT | CCACTCGCAAAGAAGCCTCCAA |  |
| *Bhi08G000682* | GGGATTGCGGACGATTTGAAGC | ATGACCGGAATCCTGGGAGTTG |  |
| *Bhi01G002439* | GGCTGTGAGGAATATGGCGTGT | TGAGGTTCTGGACATGGCGGAT | Gibberellin biosynthetic process |
| *Bhi11G000649* | GCCTTGGCTCTGTCTTGCTCTT | GGCTTAGTTGCCACTTCCTCCA | Response to gibberellin |
| *Bhi07G001018* | ACGACAGTGAGGGTGAGGTGAA | CGCCAAGCACAGCAGTCCATAT | Regulation of abscisic acid-activated signaling pathway |
| *Bhi09G001921* | CTGCCGTTCCAAGAGGTTGTCT | TGCCAATGCAGCCTGGAGATCA |  |
| *Bhi01G001743* | ATCCGCTGATGTGTTCCGTTGG | TTGGGTGTGGGTAGTGGCAGTT | Regulation of abscisic acid-activated signaling pathway |
| *Bhi12G000499* | TGAAGCTGACCGTCGTCTACCT | CAGCAGCATTCTCCCTGTGCAA |  |
| *Bhi12G002133* | CTTGGGCACAGGCAGTTCAGAA | CGCCTTCCTCTTTGTCGCCATT |  |


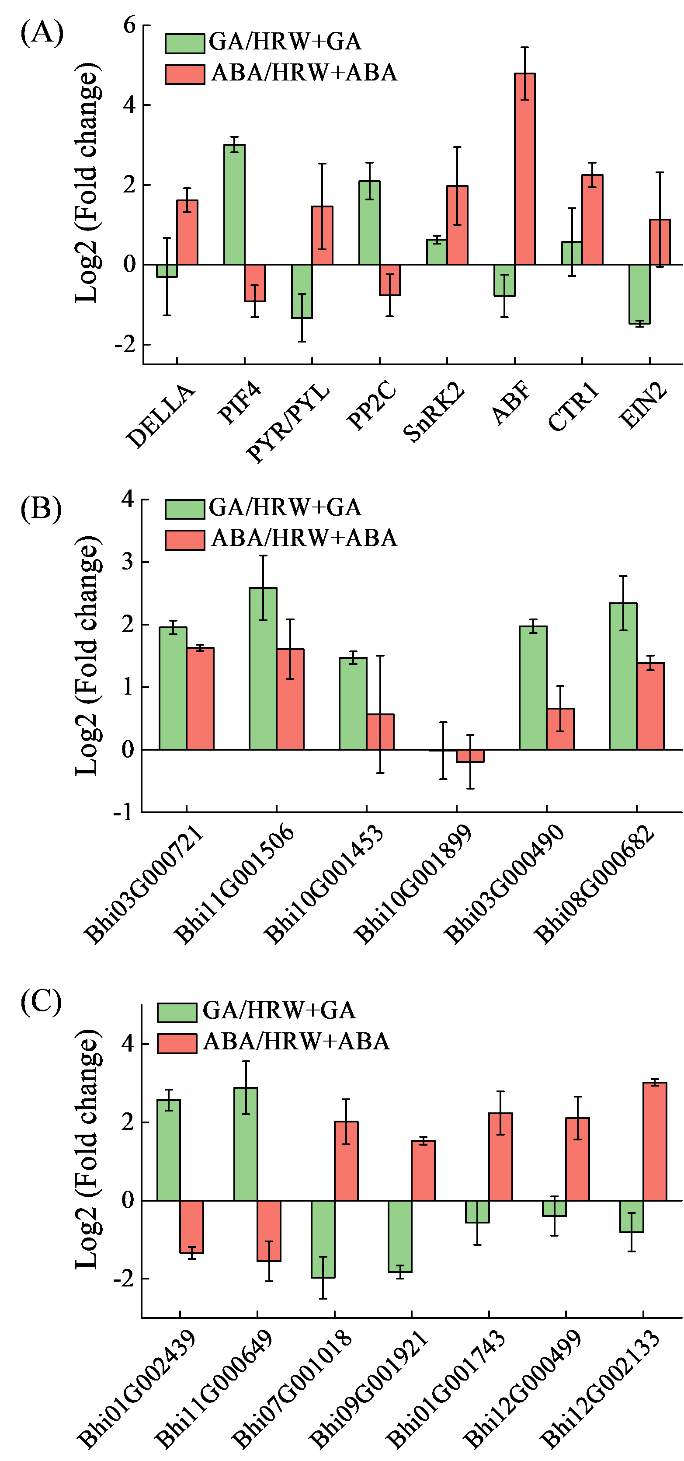


**Supplemental Figure 1** Expression analysis of DEGs via qRT-PCR in GA vs HRW+GA and ABA vs HRW+ABA group. (A) Expression analysis of genes associated to GA, ABA, and ethylene signal pathways. (B) Expression analysis of genes associated to inositol phosphate metabolism. (C) Expression analysis of genes associated to gibberellin biosynthetic process (*Bhi01G002439*), response to gibberellin (*Bhi11G000649*), and regulation of abscisic acid-activated signaling pathway. The wax gourd *BhiUBQ* (*Bhi10G000739*) gene was set as an internal control. Data of figure are the means of Log_2_ Fold Change values corresponding to each gene in a group.
